# Morphological, calorific and nutritive characteristics of 656 freshwater invertebrates taxa

**DOI:** 10.3897/BDJ.9.e70214

**Published:** 2021-10-08

**Authors:** Axelle Moreau, Christine Dupuy, Pierrick Bocher, Sébastien Farau

**Affiliations:** 1 Fédération Départementale des Chasseurs de la Vendée, Les Minées, Route de Château Fromage, 85010 La Roche-sur-Yon, France Fédération Départementale des Chasseurs de la Vendée, Les Minées, Route de Château Fromage 85010 La Roche-sur-Yon France; 2 Littoral Environnement et Sociétés (LIENSs), UMR 7266, CNRS-La Rochelle University, 17000 La Rochelle, France Littoral Environnement et Sociétés (LIENSs), UMR 7266, CNRS-La Rochelle University 17000 La Rochelle France

**Keywords:** freshwater invertebrates, invertebrate biometry, gross energy, protein, lipid, fiber, nitrogen free extract, ash, water

## Abstract

**Background:**

The Freshwater Animal Diversity Assessment (FADA) project estimated that freshwater animal species represent 9.5% of the 1.2 million species described. Knowing that freshwater represents only 0.01% of the earth's surface, these wetlands are suitable habitats for a great part of the world's total biodiversity. However, it has been shown that there is a lack of knowledge on these species, including freshwater invertebrates. Nevertheless, they play a key role in the majority of freshwater ecosystems and in their foodweb networks. Freshwater invertebrates are the food resource of many species, such as fish and birds. The knowledge of their morphological, energetic and nutritive characteristics allows a better understanding of their selection by predators (size, energy intake etc.), but also leads to the improvement of wetland management. Although information about freshwater invertebrates exists in literature, they are generally heterogeneous, dispersed and difficult to collect. To facilitate the accessibility of these data and, thus, optimise and accelerate research projects including freshwater invertebrates, we propose a literature review describing 14 morphological and nutritive characteristics (size, dry weight, gross energy, crude protein etc.) for 656 taxa of freshwater invertebrates.

**New information:**

This dataset is a review from 104 publications from 1935 to 2020, compiling 14 characteristics when available (size, dry weight, gross energy, crude protein etc.) for 656 taxa of freshwater invertebrates.

## Introduction

In twenty years, the estimated world’s total number of species has increased from 3 to 30 million (May 1990) to between 3 and 100 million (May 2010). Currently, 1.2 million species have been listed, but it was estimated that about 86% of terrestrial species and 91% of oceanic species are still to be described (Mora et al. 2011). Invertebrates would represent 60% of known species and three quarters of new species identified each year (Chapman 2009). Occupying the majority of habitats, they could potentially represent a significant part of the global biomass (Ellwood and Foster 2004). They play a key role in most trophic webs (Vanni 2002, Hornung and Foote 2006, McCarthy et al. 2009) and some are even good indicators of the environmental quality (Stork and Eggleton 1992, Hodkinson and Jackson 2005).

Wetlands are amongst the most impacted environments by climate change (Dawson et al. 2003, Erwin 2009). They have particularly declined worldwide, losing at least 50% of their surface since the beginning of the 19th century (Finlayson et al. 1999, Davidson 2014, Gardner et al. 2015). However, some species depend directly on these habitats, such as fish (Gilinsky 1984, Diehl 1992, Garvey et al. 1994, Bouffard and Hanson 2006, McCarthy et al. 2009) or birds (e.g. Bolduc and Afton 2004, Taft and Haig 2005, Ma et al. 2010), particularly because of the available food resources, such as freshwater invertebrates (Covich et al. 1999). The links between freshwater invertebrates and waterbirds were examined (Goss-Custard 1977, Colwell and Landrum 1993, Lillie and Evrard 1994, Weber and Haig 1997, Brodmann and Reyer 1999, Elmberg et al. 2000, Halse et al. 2000, Arzel et al. 2009, Guareschi et al. 2015), but remain insufficiently explored (Sanders 2000, Prather et al. 2013). The lack of knowledge about freshwater invertebrate abundance and diversity is, therefore, an important issue for the conservation of the species living in these environments, but also for their habitats management (Vanni 2002). Knowing the species that contribute to this diversity, as well as their morphological characteristics, energetic value or nutritional composition (Fredrickson and Reid 1988, Zwarts and Blomert 1992, Davis and Smith 2001), would improve the understanding of the prey-predator interactions (Nudds and Bowlby 1984, Anderson and Smith 2000). For example, waterfowl feed on aquatic invertebrates and, consequently, the abundance, accessibility, size or even the energetic values of these foods affect the use of foraging habitats by waterfowl (Ma et al. 2010).

Invertebrates are very sensitive to environmental variations, included climate change (Lawrence and Soame 2004, Prather et al. 2013, Khamis et al. 2014) and affect finally ecosystem functions and associated ecosystem services (Lavelle et al. 2006, Prather et al. 2013, Khamis et al. 2014). Some of them are also very sensitive to the water physico-chemical variations and are indicators of its quality, especially since beginning of anthropogenic pollution (Gaufin and Tarzwell 1952, Gaufin and Tarzwell 1956, Hellawell 1986, Dallinger 1994). Obtaining knowledge on freshwater invertebrates is, therefore, essential for the habitat and species conservation (Vicente 2010), but also to answer the global change (Strayer 2010, Collier et al. 2016).

Studies in literature were more specifically focused on knowledge of the orders and families, in particular, according to their stages of development (larvae, nymphs, adults) and their sizes, but it appears as still incomplete and insufficient (Vicente 2010, Strayer and Dudgeon 2010, Collier et al. 2016). If information exists, it is generally heterogeneous, scattered or restricted to local journals and, therefore, not widely distributed (Strayer 2006, Balian et al. 2008b, Strayer and Dudgeon 2010, Appeltans et al. 2012), especially when the interest is on other factors, such as the individuals weight, the energetic value or the nutritional composition (proteins, lipids etc.). Even if some books or publications have a high level of details (Cumminns and Wuycheck 1971, Nudds and Bowlby 1984, Anderson and Smith 2000, James et al. 2012), none includes all information about these physical, nutritive or energetic characteristics, placing limits to the progress of some studies. Indeed, the skills of researchers or managers do not always include know-how in taxa or species identification (Krieger 1992).

Thus, the available information in literature is generally uncommon (Strayer 2006, James et al. 2012) and/or difficult to collect (Krieger 1992). The solution for acquiring these data may be to characterise all the taxa detected and collected. However, this option raises problems related to the availability of technological tools, time (taxa collection, sorting and identification, when this skill is available, are long) (James et al. 2012) or financial (Krieger 1992, Strayer 2006) because of the human resources and the material to be mobilised. These points constitute obstacles to the progress of some studies or even points of renunciation.

In order to facilitate the accessibility of these data and, thus, promote research projects on the importance of freshwater invertebrates in wetland ecosystems, we propose a literature review of the main biological characteristics of all freshwater invertebretates available in literature up to 2020.

## Geographic coverage

### Description

This literature review concerns the worldwide freshwater wetlands (lakes, rivers, marshes, temporary and permanent ponds etc.).

## Taxonomic coverage

### Description

This dataset describes 656 taxa of freshwater invertebrates (Table 1).

### Taxa included

**Table:** 

Rank	Scientific Name	
phylum	Annelida	
subclass	Hirudinida	
genus	*Haemopis*	
family	Hirudinidae	
genus	*Macrobdella*	
family	Erpobdellidae	
species	*Dinadubia*	
genus	*Erpobdella*	
species	*Erpobdellaobscura*	
species	*Erpobdellaoctoculata*	
species	*Erpobdellapunctata*	
family	Glossiphoniidae	
species	*Glossiphoniacomplanata*	
species	*Helobdellastagnalis*	
species	*Theromyzonrude*	
family	Piscicolidae	
subclass	Oligochaeta	
family	Naididae	
species	*Aulodriluspigueti*	
species	*Branchiodrilussemperi*	
species	*Derolimosa*	
genus	*Limnodrilus*	
family	Opistocystidae	
family	Tubificidae	
genus	*Tubifex*	
family	Lumbriculidae	
species	*Lumbriculusvariegatus*	
species	*Stylodrilusheringianus*	
class	Polychaeta	
family	Nereidae	
family	Hydrachnidiae	
genus	*Limnochares*	
subclass	Aranae	
order	Anostraca	
species	*Branchinectapaludosa*	
species	*Streptocephalussealii*	
order	Diplostraca	
suborder	Cladocera	
species	*Acantholeberiscurvirostris*	
family	Bosminidae	
genus	*Bosmina*	
species	*Bosminacoregoni*	
species	*Bosminahagmanni*	
species	*Bosminalongirostris*	
species	*Bythotrepheslongimanus*	
family	Chydoridae	
genus	*Alona*	
species	*Alonaaffinis*	
species	*Alonacostata*	
species	*Alonarectangula*	
species	*Alonellaexigua*	
species	*Alonellanana*	
species	*Chydorussphaericu*s	
species	*Eurycercuslamellatus*	
species	*Graptoleberistestudinaria*	
species	*Leydigialeydigi*	
species	*Pleuroxusaduncus*	
species	*Acroperusharpae*	
species	*Dunhevediacrassa*	
family	Daphniidae	
species	*Ceriodaphniaquadrangula*	
species	*Ceriodaphniareticulata*	
species	*Ceriodaphniasilvestrii*	
genus	*Daphnia*	
species	*Daphniaambigua*	
species	*Daphniacristata*	
species	*Daphniacucullata*	
species	*Daphniadubia*	
species	*Daphniagaleata*	
species	*Daphniagessneri*	
species	*Daphniahyalina*	
species	*Daphniamagna*	
species	*Daphniapulex*	
species	*Diaphanosomaspinulosum*	
species	*Moinadubia*	
species	*Scapholeberismucronata*	
species	*Simocephalusvetulus*	
species	*Holopediumgibberum*	
family	Leptodoridae	
species	*Leptodorakindtii*	
species	*Leptodorakindtii*	
species	*Moinamacrocopa*	
species	*Moinamicrura*	
species	*Moinamongolica*	
species	*Polyphemuspediculus*	
species	*Diaphanosomabrachyurum*	
species	*Diaphanosomapauscispinosum*	
family	Cyzicidae	
species	*Caenestheriellasetosa*	
family	Leptestheriidae	
family	Lynceidae	
species	*Lynceusbrachyurus*	
order	Notostraca	
species	*Lepiduruscouesii*	
species	*Chirocephalopsisbundyi*	
order	Amphipoda	
genus	*Eogammarus*	
species	*Chelicorophiumcurvispinum*	
genus	*Corophium*	
species	*Crangonyxgracilis*	
species	*Crangonyxrichmondensis*	
species	*Gammaracanthuslacustris*	
family	Gammaridae	
genus	*Gammarus*	
species	*Gammarusfasciatus*	
species	*Gammarusfossarum*	
species	*Gammaruslacustris*	
species	*Gammaruspulex*	
species	*Gammarusroeseli*	
genus	*Hyalella*	
species	*Hyalellaazteca*	
species	*Hyalellacurvispina*	
species	*Pallasiolaquadrispinosa*	
species	*Paracalliopefluviatilis*	
family	Pontogammaridae	
species	*Monoporeiaaffinis*	
species	*Pontoporeiahoyi*	
family	Talitridae	
order	Decapoda	
genus	*Aegla*	
species	*Aeglaneuquensis*	
family	Astacidae	
family	Cambaridae	
species	*Faxoniusvirilis*	
species	*Atyaephyradesmarestii*	
species	*Macrobrachiumrosenbergii*	
species	*Palaemonlamarrie*	
species	*Samastacusspinifrons*	
genus	*Orconectes*	
order	Isopoda	
genus	*Gnorimosphaeroma*	
family	Asellidae	
genus	*Asellus*	
species	*Asellusaquaticus*	
species	*Caecidotearacovitzai*	
species	*Lirceuslineatus*	
species	*Proasellusmeridianus*	
family	Sphaeromatidae	
species	*Austridoteaannectens*	
family	Mysidae	
species	*Mysisrelicta*	
species	*Tenagomysischiltoni*	
subclass	Copepoda	
species	*Limnocalanusmacrurus*	
species	*Acanthocyclopsrobustus*	
species	*Mesocyclopsogunnus*	
family	Diaptomidae	
species	*Argyrodiaptomusazevedoi*	
species	*Diaptomusarcticus*	
species	*Diaptomusclavipes*	
species	*Diaptomusleptopus*	
species	*Diaptomusminutus*	
species	*Diaptomussiciloides*	
species	*Eudiaptomusgracilis*	
species	*Eudiaptomuspadanus*	
species	*Heliodiaptomuscinctus*	
species	*Notodiaptomuscearensis*	
species	*Notodiaptomusevaldus*	
species	*Notodiaptomusiheringi*	
species	*Eurytemoraaffinis*	
order	Cyclopoida	
family	Cyclopidae	
genus	*Cyclops*	
species	*Cyclopsabyssorum*	
species	*Cyclopsbicuspidatus*	
species	*Macrocyclopsalbidus*	
species	*Mesocyclopsedax*	
species	*Thermocyclopshyalinus*	
order	Harpacticoida	
class	Ostracoda	
family	Cyprididae	
species	*Stenocyprismalcolmsonii*	
order	Collembola	
order	Coleoptera	
family	Dytiscidae	
species	*Agabusbifarius*	
species	*Colymbetessculptilis*	
species	*Cybistertripunctatus*	
genus	*Dytiscus*	
species	*Dytiscusmarginalis*	
species	*Rhantusfrontalis*	
family	Gyrinidae	
species	*Gyrinusmaculiventris*	
species	*Enochrushamiltoni*	
species	*Galerucellanymphaeae*	
family	Curculionidae	
family	Elmidae	
species	*Ancronyxvariegata*	
genus	*Austrelmis*	
species	*Macronychusglabratus*	
genus	*Promoresia*	
genus	*Stenelmis*	
genus	*Optioservus*	
family	Hydrophilidae	
genus	*Cylomissus*	
species	*Enochruscarinatus*	
species	*Hydrophilusolivaceus*	
species	*Tropisternussetiger*	
genus	*Ectopria*	
genus	*Psephenus*	
species	*Anchytarsusbicolor*	
genus	*Hydrocyphon*	
order	Diptera	
family	Athericidae	
genus	*Atherix*	
species	*Atherixibis*	
genus	*Dasyoma*	
family	Dolichopodidae	
family	Empididae	
family	Ephydridae	
family	Stratiomyiidae	
genus	*Caloparyphus*	
species	*Hedriodiscustruquii*	
genus	*Stratiomys*	
family	Syrphidae	
genus	*Eristalis*	
family	Tabanidae	
genus	*Chrysops*	
genus	*Tabanus*	
suborder	Nematocera	
family	Blephariceridae	
genus	*Edwardsina*	
genus	*Blepharicera*	
family	Ceratopogonidae	
genus	*Palpomyia*	
genus	*Chaoborus*	
species	*Chaoborusflavicans*	
family	Chironomidae	
genus	*Ablabesmyia*	
species	*Ablabesmyiapulchripennis*	
genus	*Chironomus*	
species	*Chironomusmodestus*	
species	*Chironomusplumosus*	
species	*Chironomustentans*	
species	*Chironomuszealandicus*	
genus	*Cladopelma*	
genus	*Cladotanytarsus*	
genus	*Coelotanypus*	
genus	*Cricotopus*	
genus	*Dicrotendipes*	
species	*Dicrotendipestenuiforceps*	
species	*Endochironomusalbipennis*	
genus	*Glyptotendipes*	
species	*Glyptotendipesbarbipes*	
species	*Heterotrissocladiusgrimshawi*	
genus	*Metriocnemus*	
species	*Microtendipespedellus*	
genus	*Pagastiella*	
species	*Parakiefferiellanigra*	
genus	*Paratrichocladius*	
genus	*Paucispinigera*	
species	*Phaenopsectraalbescens*	
genus	*Polypedilum*	
species	*Polypedilumuncinatum*	
genus	*Procladius*	
genus	*Psectrocladius*	
species	*Psectrocladiussemicirculatus*	
species	*Psectrotanypusdyari*	
genus	*Tanytarsus*	
species	*Tanytarsuslewisi*	
genus	*Zavrelia*	
family	Culicidae	
species	*Aedesaegypti*	
species	*Aedescanadensis*	
species	*Culexpipiens*	
family	Dixidae	
genus	*Dixa*	
family	Limoniidae	
species	*Aphrophilaneozelandica*	
genus	Hexatoma	
genus	*Lipsothrix*	
genus	*Molophilus*	
genus	*Dicranota*	
genus	*Pedicia*	
genus	*Pilaria*	
family	Psychodidae	
genus	*Clogmia*	
genus	*Psychoda*	
family	Sciaridae	
family	Simuliidae	
genus	*Austrosimulium*	
genus	*Simulium*	
family	Tipulidae	
genus	*Hexatoma*	
genus	*Tipula*	
species	*Tipulaabdominalis*	
order	Ephemeroptera	
genus	*Baetisca*	
family	Caenidae	
genus	*Caenis*	
species	*Caenisdiminuta*	
species	*Caenishoraria*	
species	*Caenisrobusta*	
family	Ephemerellidae	
species	*Cincticostellanigra*	
species	*Cincticostellaorientalis*	
genus	*Drunella*	
species	*Drunellabasalis*	
genus	*Ephemerella*	
species	*Ephemerellaaurivillii*	
species	*Eurylophellatemporalis*	
genus	*Serratella*	
species	*Teloganopsispunctisetae*	
family	Ephemeridae	
genus	*Ephemera*	
species	*Ephemerajaponica*	
species	*Ephemerastrigata*	
genus	*Blasturus*	
genus	*Deleatidium*	
species	*Habrophlebiavibrans*	
genus	*Leptophlebia*	
species	*Leptophlebiavespertina*	
genus	*Meridialaris*	
species	*Nousiabella*	
genus	*Paraleptophlebia*	
species	*Paraleptophlebiawestoni*	
species	*Penaphlebiachilensis*	
genus	*Zephlebia*	
species	*Ephoronalbum*	
family	Potamanthidae	
genus	*Tricorythodes*	
genus	*Ameletus*	
species	*Ameletopsisperscitus*	
species	*Chiloportereatoni*	
family	Ametropodidae	
family	Baetidae	
genus	*Acentrella*	
species	*Acentrellagnom*	
species	*Baetiellajaponica*	
genus	*Baetis*	
species	*Baetisthermicus*	
genus	*Callibaetis*	
species	*Centroptilumluteolum*	
species	*Cloeondipterum*	
genus	*Heterocloeon*	
genus	*Pseudocloeon*	
species	*Coloburiscushumeralis*	
family	Heptageniidae	
genus	*Cinygmula*	
genus	*Ecdyonurus*	
species	*Ecdyonurusdispar*	
genus	*Epeorus*	
species	*Epeorusdispar*	
species	*Epeorusikanonis*	
species	*Epeoruslatifolium*	
species	*Epeoruspleuralis*	
genus	*Heptagenia*	
species	*Kageroniafuscogrisea*	
genus	*Leucrocuta*	
species	*Maccaffertiummeririvulanum*	
genus	*Nixe*	
genus	*Rhithrogena*	
species	*Stenacroncarolina*	
species	*Stenacroninterpunctatum*	
genus	*Stenonema*	
species	*Stenonemamodestum*	
genus	*Isonychia*	
species	*Isonychiajaponica*	
genus	*Nesameletus*	
genus	*Metamonius*	
species	*Siphloniscaaerodromia*	
genus	*Siphlonurus*	
order	Hemiptera	
species	*Aphelocheirusvittatus*	
family	Belostomatidae	
species	*Lethocerusdeyrollei*	
species	*Lethocerusindicus*	
family	Corixidae	
species	*Callicorixaaudeni*	
species	*Corisellamercenaria*	
species	*Corixapunctata*	
species	*Cymatiaamericana*	
family	Gerridae	
family	Nepidae	
species	*Laccotrephesjaponensis*	
species	*Laccotrephesmaculatus*	
species	*Laccotrephesruber*	
family	Notonectidae	
species	*Notonectaglauca*	
species	*Notonectakirbyi*	
species	*Neopleastriola*	
species	*Microveliamacgregori*	
order	Hymenoptera	
order	Lepidoptera	
family	Pyralidae	
genus	*Petrophila*	
order	Megaloptera	
family	Corydalidae	
species	*Archichauliodesdiversus*	
species	*Corydaluscornutus*	
species	*Nigroniaserricornis*	
species	*Parachauliodescontinentalis*	
species	*Protohermesgrandis*	
family	Sialidae	
genus	*Sialis*	
species	*Sialisaequalis*	
species	*Sialisitasca*	
species	*Sialislutaria*	
species	*Sialisvelata*	
order	Neuroptera	
order	Odonata	
suborder	Anisoptera	
family	Aeshnidae	
genus	*Aeshna*	
species	*Aeshnainterrupta*	
species	*Cordulegastermaculata*	
species	*Corduliaaenea*	
genus	*Epitheca*	
species	*Epithecacynosura*	
species	*Epithecasemiaquea*	
species	*Neurocorduliamolesta*	
genus	*Dromogomphus*	
family	Gomphidae	
genus	*Gomphus*	
species	*Lanthusvernalis*	
species	*Megalogomphussuperbus*	
species	*Progomphusobscurus*	
species	*Sieboldiusalbardae*	
family	Libellulidae	
species	*Celithemisfasciata*	
species	*Crocothemisservilia*	
species	*Erythemissimplicicollis*	
species	*Ladonadeplanata*	
genus	*Libellula*	
species	*Plathemislydia*	
genus	*Procordulia*	
species	*Sympetruminternum*	
suborder	Zygoptera	
family	Calopterygidae	
genus	*Calopteryx*	
family	Coenargionidae	
genus	*Argia*	
species	*Argiavivida*	
species	*Coenagrionangulatum*	
species	*Coenagrionresolutum*	
species	*Enallagmaboreale*	
genus	*Ischnura*	
species	*Ischnuraelegans*	
genus	*Xanthocnemis*	
family	Lestidae	
species	*Lestescongener*	
species	*Lestesdisjunctus*	
species	*Lestesdryas*	
species	*Lestesmalabaricus*	
species	*Lestesrectangularis*	
order	Orthoptera	
order	Plecoptera	
species	*Austroperlacyrene*	
species	*Klapopteryxkuscheli*	
species	*Stenoperlaprasina*	
species	*Antarctoperlamichaelseni*	
species	*Aubertoperlailliesi*	
species	*Limnoperlajaffueli*	
species	*Notoperlaarchiplatae*	
species	*Notoperlopsisfemina*	
species	*Potamoperlamyrmidon*	
species	*Senzilloidespanguipulli*	
genus	*Zelandobius*	
genus	*Zelandoperla*	
genus	*Allocapnia*	
species	*Allocapniarickeri*	
family	Chloroperlidae	
genus	*Sweltsa*	
genus	*Leuctra*	
family	Nemouridae	
genus	*Amphinemura*	
species	*Amphinemuradelosa*	
species	*Amphinemurawui*	
species	*Prostoiacompleta*	
genus	*Tallaperla*	
genus	*Acroneuria*	
species	*Acroneuriaabnormis*	
species	*Acroneuriaevoluta*	
species	*Agnetinacapitata*	
species	*Beloneuriageorgiana*	
genus	*Caroperla*	
species	*Eccopturaxanthenes*	
species	*Kamimuriauenoi*	
species	*Kempnyelagenualis*	
genus	*Kiotina*	
genus	*Neoperla*	
species	*Neoperlaclymene*	
genus	*Paragnetina*	
species	*Paragnetinakansensis*	
genus	*Perla*	
genus	*Perlesta*	
species	*Perlestaplacida*	
species	*Perlinelladrymo*	
species	*Pictetoperlagayi*	
family	Perlodidae	
species	*Clioperlaclio*	
genus	*Isogenus*	
genus	*Isoperla*	
species	*Isoperlabilineata*	
species	*Isoperlanamata*	
species	*Isoperlasignata*	
species	*Malirekushastatus*	
genus	*Perlodes*	
genus	*Stavsolus*	
species	*Pteronarcysdorsata*	
species	*Pteronarcysscotti*	
genus	*Strophopteryx*	
species	*Strophopteryxlimata*	
genus	*Taeniopteryx*	
species	*Taeniopteryxlita*	
order	Trichoptera	
family	Ecnomidae	
species	*Ecnomustenellus*	
family	Hydropsychidae	
genus	*Aoteapsyche*	
genus	*Cheumatopsyche*	
species	*Cheumatopsycheinfascia*	
genus	*Hydropsyche*	
species	*Hydropsychealbicephala*	
species	*Hydropsychedissimulata*	
species	*Hydropsycheelissoma*	
species	*Hydropsychepellucidula*	
species	*Hydropsychesparna*	
species	*Hydropsycheincommoda*	
species	*Macronemapseudoneura*	
species	*Macrostemumcarolina*	
genus	*Smicridea*	
genus	*Chimarra*	
genus	*Dolophilodes*	
family	Polycentropodidae	
genus	*Cyrnus*	
species	*Cyrnuscrenaticornis*	
species	*Cyrnusflavidus*	
species	*Cyrnustrimaculatus*	
genus	*Neureclipsis*	
genus	*Polycentropus*	
species	*Polycentropusflavomaculatus*	
family	Psychomyiidae	
species	*Lypediversa*	
species	*Tinodeswaeneri*	
species	*Stenopsychemarmorata*	
species	*Stenopsychesauteri*	
genus	*Brachycentrus*	
species	*Brachycentrusetowahensis*	
genus	*Micrasema*	
species	*Micrasemaquadriloba*	
genus	*Phylloicus*	
species	*Olingaferedayi*	
genus	*Pycnocentrodes*	
species	*Goerajaponica*	
genus	*Helicopsyche*	
genus	*Lepidostoma*	
family	Leptoceridae	
genus	*Athripsodes*	
species	*Athripsodesaterrimus*	
genus	*Ceraclea*	
species	*Mystacideslongicornis*	
species	*Mystacidesnigra*	
genus	*Nectopsyche*	
genus	*Oecetis*	
species	*Triaenodestardus*	
family	Limnephilidae	
genus	*Anabolia*	
species	*Anaboliabimaculata*	
species	*Anaboliafurcata*	
species	*Dicosmoecusjozankeanus*	
species	*Hydatophylaxfestivus*	
species	*Ironoquiaparvula*	
genus	*Limnephilus*	
species	*Limnephilusjanus*	
species	*Limnephilusrhombicus*	
species	*Philarctusquaeris*	
species	*Potamophylaxcingulatus*	
species	*Pycnopsycheguttifera*	
species	*Pycnopsychelepida*	
species	*Pycnopsycheluculenta*	
species	*Pycnopsychescabripennis*	
species	*Molannaangustata*	
genus	*Psilotreta*	
family	Phryganeidae	
species	*Agrypniaobsoleta*	
genus	*Phryganea*	
species	*Phryganeacinerea*	
genus	*Ptilostomis*	
family	Sericostomatidae	
species	*Agarodeslibalis*	
species	*Fattigiapele*	
species	*Parasericostomaovale*	
genus	*Glossosoma*	
species	*Glossosomaaltaicum*	
species	*Glossosomanigrior*	
species	*Glossosomaussuricum*	
family	Hydrobiosidae	
genus	*Neoatopsyche*	
genus	*Rheochorema*	
genus	*Cailloma*	
family	Hydroptilidae	
genus	*Hydroptila*	
genus	*Orthotrichia*	
genus	*Oxyethira*	
genus	*Paroxyethira*	
family	Rhyacophilidae	
genus	*Rhyacophila*	
species	*Rhyacophilakawamurae*	
species	*Rhyacophilavao*	
family	Corbiculidae	
species	*Dreissenapolymorpha*	
family	Sphaeriidae	
genus	*Pisidium*	
species	*Pisidiumcasertanum*	
family	Hyriidae	
family	Unionidae	
species	*Anodontacataracta*	
species	*Elliptiocomplanata*	
species	*Leptodeaochracea*	
species	*Parreysiafavidens*	
species	*Parreysiakhadakvaslaensis*	
class	Gastropoda	
family	Viviparidae	
species	*Bellamyadissimilis*	
family	Amnicolidae	
species	*Amnicolastenothyroides*	
family	Bithyniidae	
species	*Bithyniatentaculata*	
family	Hydrobiidae	
species	*Potamopyrgusantipodarum*	
family	Bithyniidae	
species	*Elimiacahawbensis*	
species	*Elimiacarinifera*	
species	*Elimiafascinans*	
species	*Elimiavariata*	
species	*Pleuroceracanaliculatum*	
family	Thiaridae	
species	*Melanoidestuberculata*	
family	Valvatidae	
species	*Valvatacristata*	
species	*Succineaovalis*	
family	Acroloxidae	
species	*Chilinagibbosa*	
species	*Chilinapatagonica*	
family	Lymnaeidae	
species	*Lymnaeastagnalis*	
species	*Radixperegra*	
family	Physidae	
family	Planorbidae	
genus	*Ancylus*	
species	*Anisusvortex*	
genus	*Gyraulus*	
species	*Gyraulusalbus*	
species	Gyrauluscrista	
species	*Hippeutiscomplanatus*	
species	*Planorbisindicus*	
phylum	Platyhelminthes	
class	Rhabditophora	
order	Tricladida	
species	*Dendrocoelumlacteum*	
species	*Curaforemanii*	
species	*Dugesiatigrina*	
genus	*Girardia*	
species	*Girardiatigrina*	
species	*Polycelisnigra*	

## Temporal coverage

### Notes

The oldest publication was published in 1935 and the most recent in 2020.

## Usage licence

### Usage licence

Creative Commons Public Domain Waiver (CC-Zero)

## Data resources

### Data package title

Freshwater invertebrates characteristics data

### Number of data sets

1

### Data set 1.

#### Data set name

Freshwater invertebrates characteristics data

#### Data format

.txt

#### Number of columns

26

#### Description

This dataset describes 14 characteristics (size, dry weight, gross energy, crude protein etc.) for 656 taxa of freshwater invertebrates (Suppl. material 1). Headers corresponding to variable names are included as the first row in the data file. Each characteristic is subdivided into two categories, "value" corresponding to the value of the variable with sometimes a note corresponding to the comment associated with the value ("WS" for "with shell" and "SR" for "shell removed") and "reference" corresponding to the literature reference (Suppl. material 2). The datasets are deposited in Dryad (doi: 10.5061/dryad.j3tx95xfg).

**Data set 1.** 

Column label	Column description
ID	Unique number corresponding to a described taxa
Phylum	Identification of taxa (Phylum)
Subphylum	Identification of taxa (Subphylum)
Class	Identification of taxa (Class)
Subclass	Identification of taxa (Subclass)
Order	Identification of taxa (Order)
Suborder	Identification of taxa (Suborder)
Family	Identification of taxa (Familly)
Genus	Identification of taxa (Genus)
Species	Identification of taxa (Genus + Species)
Taxa	Identification of taxa (Taxa)
LifeStage	The life stage of the taxa, i.e. "Larvae", "Larvae I", "Larvae II", "Larvae III", "Larvae IV", "Larvae V", "Larvae VI", "Nymph", "Pupae" or "Adult".
TL (value)	Total length in millimetres
HW/SW (value)	Head width / Shell width in millimetres
BL/SL (value)	Body length / Shell length in millimetres
WW (value)	Wet weight in milligrams
DW (value)	Dry weight in milligrams
GE-WW (value)	Gross energy in cal/g of wet weight
GE-DW (value)	Gross energy in cal/g of dry weight
GE-AFDW (value)	Gross energy in cal/g of ash free dry weight
Protein (value)	Crude protein in % of aggregate dry weight
Lipid (value)	Crude lipid in % of aggregate dry weight
Fibre (value)	Crude fibre in % of aggregate dry weight
NFE (value)	Nitrogen Free Extract in % of aggregate dry weight
Ash (value)	Ash in % of aggregate dry weight
Water (value)	Water in % of aggregate dry weight

## Additional information

### Steps of database building

1. **Classification and characteristics of freshwater invertebrates**: A literature review of the different morphological, calorific and nutritive characteristics of freshwater invertebrates was established. They were classified from phylum to species from three manuals of references (Balian et al. 2008a, Thorp and Covich 2009, Thorp and Rogers 2011). In this review, 104 scientific publications were used and 14 criteria were described: total length, head width or shell width, body length or shell length, wet weight, dry weight, gross energy of wet weight, gross energy of dry weight, gross energy of ash free dry weight, crude protein, crude lipid, crude fibre, nitrogen free extract, proportion of ash and water.
2. **Referencing and retranscription of literature values**: Publications presenting original data or non-published data from another study were cited. Within publications, the values of the different criteria were represented heterogeneously (e.g. different measure units, taxonomic level). Due to this heterogeneity, all values were transformed to obtain a homogeneous set of measurement units and taxonomic level (i.e. μm in mm, µg in mg, kcal/g in cal/g, J/g in cal/g (1 J = 0.239006 cal), kJ/g in cal/g).

### Prospects for use

Scientific literature shows that abundance of food resource is one of the first things that the animal ecologists measure when they want to understand the species they are studying, whether it is individual behaviour, reproduction or even population dynamics (Newton 1998). Thus, estimates of biomass or the calorific equivalent of freshwater organisms are necessary in the study of the food ecology of fish, amphibians or even birds. For example, the availability of food is often considered to be a fundamental factor affecting the migration and reproduction of animals, especially birds. For example, Anatidae conventionally have a diet dominated by seeds and plant matter in winter, but aquatic invertebrates dominate in spring and summer (Krapu and Reinecke 1994). Thus, the annual migration of Anatidae between the wintering and breeding site is often explained by the exploitation of abundant food resources at higher latitudes to increase breeding success (Berthold et al. 2003). In addition, many scientists agree that migration is programmed according to local peaks of food abundance at successive stopover sites in order to feed during migration and prepare for reproduction (Ankney et al. 1991, Drent and Daan 2002). After prenuptial migration, the majority of surface ducks must efficiently replenish fat and protein stores because egg formation and incubation are expensive processes (Alisauskas and Ankney 1992). Consequently, the abundance of food appears to be a decisive factor in the choice of breeding habitat (Pöysä et al. 2000). For all these reasons, the abundance, biomass and nutritional value of invertebrates are essential in studying habitat choice, reproductive success and annual cycles of dabbling ducks (Batt et al. 1992). In addition, prey size is essential to understand the food ecology of the organisms studied. For example, the Northern Shoveler has a spatula-shaped beak made up of many lamellae. This physical characteristic allows it to select only small prey, giving it a specific food niche. Thus, it is important to know the size of the prey available in order to understand the use of a site by this species.

The abundance, biomass and nutritional value of freshwater invertebrates are, therefore, essential in the study of predatory of freshwater invertebrates (Towers et al. 1994). The most common method for determining the wet or dry weight of aquatic invertebrates is to directly weigh individual specimens (Smock 1980). However, this approach often takes time and is prone to error if individuals were fixed (Downing 1984). Indeed, preservative often modifies the fresh mass of conserved invertebrates (Johnston and Cunjak 1999). The dry mass measurement has the disadvantage of rendering the sample unusable for further examination following the drying process (Towers et al. 1994). In this case, a table synthesising the biometric and calorific data of freshwater invertebrates is a real benefit for the ecologist. Indeed, this table will allow freshwater ecologists to estimate biomass, energy and size class of freshwater invertebrates at survey sites quickly and economically.

However, it is important to note that there is variation in calorific values due to the collection season, the diet of the organisms and the sex of the individuals (Arzel et al. 2009). In addition, there is variation in individual values because the measurements taken almost always include the intestinal contents of the organisms or because females carrying eggs should have the highest values for a given species (Cumminns 1967). Given the errors that environmentalists face, it might be more realistic to use a median or overall calorific value. For the taxon studied, this value is, thus, easily obtainable thanks to this table. Indeed, the comparison of values for the same species obtained in different laboratories should allow problems due to seasonal differences, habitat and diet to be overcome (Johnston and Cunjak 1999).

## Supplementary Material

0A5F1E0C-DBAD-5D43-946A-5C0EE36C75D8 10.3897/BDJ.9.e70214.suppl1 Supplementary material 1 Freshwater invertebrates characteristics data Data type Morphological, calorific and nutritive File: oo_582217.txt https://binary.pensoft.net/file/582217 Moreau A

92770F81-9F39-577E-B285-395B13ABFFBC 10.3897/BDJ.9.e70214.suppl2 Supplementary material 2 Reference code Data type References File: oo_551812.txt https://binary.pensoft.net/file/551812 Moreau A

## Figures and Tables

**Table 1.** Number of described taxa.

**Phylum**	**Subphylum**	**Class**	**Subclass**	**Order**	**Suborder**	**Familly**	**Genus**	**Species**
Annelida	-	Clitellata	2	2	3	10	14	14
-	-	Polychaeta	1	1	1	1	0	-
Arthropoda	Chelicerata	1	2	1	1	2	1	-
-	Crustacea	4	4	12	10	44	66	98
-	Hexapoda	2	1	13	15	98	325	209
Mollusca	-	Bivalvia	2	2	-	5	6	7
-	-	Gastropoda	3	6	1	14	17	21
Plathelminthes	-	1	1	1	1	3	5	5

## References

[B7434619] Alisauskas Ray T., Ankney C. Davison (1992). Spring Habitat Use and Diets of Midcontinent Adult Lesser Snow Geese. The Journal of Wildlife Management.

[B6160634] Anderson J. T., Smith L. M. (2000). Invertebrate response to moist‐soil management of playa wetlands. Ecological Applications.

[B7434628] Ankney C. Davison, Afton Alan D., Alisauskas Ray T. (1991). The Role of Nutrient Reserves in Limiting Waterfowl Reproduction. The Condor.

[B6160508] Appeltans Ward, Ahyong Shane T., Anderson Gary, Angel Martin V., Artois Tom, Bailly Nicolas, Bamber Roger, Barber Anthony, Bartsch Ilse, Berta Annalisa, Błażewicz-Paszkowycz Magdalena, Bock Phil, Boxshall Geoff, Boyko Christopher B., Brandão Simone Nunes, Bray Rod A., Bruce Niel L., Cairns Stephen D., Chan Tin-Yam, Cheng Lanna, Collins Allen G., Cribb Thomas, Curini-Galletti Marco, Dahdouh-Guebas Farid, Davie Peter J. F., Dawson Michael N., De Clerck Olivier, Decock Wim, De Grave Sammy, de Voogd Nicole J., Domning Daryl P., Emig Christian C., Erséus Christer, Eschmeyer William, Fauchald Kristian, Fautin Daphne G., Feist Stephen W., Fransen Charles H. J.M., Furuya Hidetaka, Garcia-Alvarez Oscar, Gerken Sarah, Gibson David, Gittenberger Arjan, Gofas Serge, Gómez-Daglio Liza, Gordon Dennis P., Guiry Michael D., Hernandez Francisco, Hoeksema Bert W., Hopcroft Russell R., Jaume Damià, Kirk Paul, Koedam Nico, Koenemann Stefan, Kolb Jürgen B., Kristensen Reinhardt M., Kroh Andreas, Lambert Gretchen, Lazarus David B., Lemaitre Rafael, Longshaw Matt, Lowry Jim, Macpherson Enrique, Madin Laurence P., Mah Christopher, Mapstone Gill, McLaughlin Patsy A., Mees Jan, Meland Kenneth, Messing Charles G., Mills Claudia E., Molodtsova Tina N., Mooi Rich, Neuhaus Birger, Ng Peter K. L., Nielsen Claus, Norenburg Jon, Opresko Dennis M., Osawa Masayuki, Paulay Gustav, Perrin William, Pilger John F., Poore Gary C. B., Pugh Phil, Read Geoffrey B., Reimer James D., Rius Marc, Rocha Rosana M., Saiz-Salinas José I., Scarabino Victor, Schierwater Bernd, Schmidt-Rhaesa Andreas, Schnabel Kareen E., Schotte Marilyn, Schuchert Peter, Schwabe Enrico, Segers Hendrik, Self-Sullivan Caryn, Shenkar Noa, Siegel Volker, Sterrer Wolfgang, Stöhr Sabine, Swalla Billie, Tasker Mark L., Thuesen Erik V., Timm Tarmo, Todaro Antonio M., Turon Xavier, Tyler Seth, Uetz Peter, van der Land Jacob, Vanhoorne Bart, van Ofwegen Leen P., van Soest Rob W. M., Vanaverbeke Jan, Walker-Smith Genefor, Walter Chad T., Warren Alan, Williams Gary C., Wilson Simon P., Costello Mark J. (2012). The magnitude of global marine species diversity. Current Biology.

[B6160496] Arzel Céline, Elmberg Johan, Guillemain Matthieu, Lepley Michel, Bosca Fabrice, Legagneux Pierre, Nogues Jean-Baptiste (2009). A flyway perspective on food resource abundance in a long-distance migrant, the Eurasian teal (*Anascrecca*). Journal of Ornithology.

[B6903269] Balian E. V., Lévêque C., Segers H., Martens K. (2008). Freshwater animal diversity assessment.

[B6160469] Balian E. V., Segers H., Lévèque C., Martens K. (2008). The freshwater animal diversity assessment: an overview of the results. Hydrobiologia.

[B7434637] Batt Bruce D. J., Anderson Michael G., Afton Alan D. (1992). Ecology and Management of Breeding Waterfowl.

[B7434611] Berthold Peter, Gwinner Eberhard, Sonnenschein Edith (2003). Avian Migration.

[B6160856] Bolduc F., Afton A. D. (2004). Relationships between wintering waterbirds and invertebrates, sediments and hydrology of coastal marsh ponds. Waterbirds: The International Journal of Waterbird Biology.

[B6160487] Bouffard Stephen H., Hanson Mark A. (2006). Fish in waterfowl marshes: waterfowl managers' perspective. Wildlife Society Bulletin (1973-2006).

[B6160838] Brodmann Paul A., Reyer Heinz-Ulrich (1999). Nestling provisioning in water pipits (*Anthusspinoletta*): Do parents go for specific nutrients or profitable prey?. Oecologia.

[B6160011] Chapman Arthur D. (2009). Numbers of living species in Australia and the World. https://www.environment.gov.au/science/abrs/publications/other/numbers-living-species/contents.

[B6160937] Collier Kevin J., Probert P. Keith, Jeffries Michael (2016). Conservation of aquatic invertebrates: concerns, challenges and conundrums: Conservation of aquatic invertebrates. Aquatic Conservation: Marine and Freshwater Ecosystems.

[B6160811] Colwell M. A., Landrum S. L. (1993). Nonrandom shorebird distribution and fine-scale variation in prey abundance. Condor.

[B6159659] Covich A. P., Palmer M. A., Crowl T. A. (1999). The role of benthic invertebrate species in freshwater ecosystem. BioScience.

[B7434586] Cumminns K. W. (1967). Calorific Equivalents for Studies in Ecological Energetics.

[B6159650] Cumminns K. W., Wuycheck J. C. (1971). Caloric equivalents for investigations in ecological energetics. International Association of Theoretical and Applied Limnology.

[B6159537] Dallinger Reinhard (1994). Invertebrate organisms as biological indicators of heavy metal pollution. Applied Biochemistry and Biotechnology.

[B6160074] Davidson Nick C. (2014). How much wetland has the world lost? Long-term and recent trends in global wetland area. Marine and Freshwater Research.

[B6160820] Davis C. A., Smith L. M. (2001). Foraging strategies and niche dynamics of coexisting shorebirds at stopover sites in the Southern Great Plains. The Auk.

[B6160892] Dawson Terry P., Berry Pam M., Kampa E. (2003). Climate change impacts on freshwater wetland habitats. Journal for Nature Conservation.

[B6160883] Diehl Sebastian (1992). Fish predation and benthic community structure: The role of omnivory and habitat complexity. Ecology.

[B7434564] Downing John A, Downing John A (1984). A Manual on Methods for the Assessment of Secondary Productivity.

[B7434533] Drent R. H., Daan S. (2002). The Prudent Parent: Energetic Adjustments in Avian Breeding. Ardea.

[B6160065] Ellwood Martin D. F., Foster William A. (2004). Doubling the estimate of invertebrate biomass in a rainforest canopy. Nature.

[B6160874] Elmberg Johan, Sjöberg Kjell, Pöysä Hannu, Nummi Petri (2000). Abundance-distribution relationships on interacting trophic levels: the case of lake-nesting waterfowl and dytiscid water beetles. Journal of Biogeography.

[B6160865] Erwin Kevin L. (2009). Wetlands and global climate change: the role of wetland restoration in a changing world. Wetlands Ecology and Management.

[B6160037] Finlayson C. M., Davidson N. C., Spiers A. G., Stevenson N. J. (1999). Global wetland inventory - current status and future priorities. Marine and Freshwater Research.

[B6160456] Fredrickson L. H., Reid F. A., Cross D. H. (1988). Waterfowl management handbook.

[B6160110] Gardner R. C., Barchiesi S., Beltrame C., Finlayson C. M., Galewski T., Harrison I., Paganini M., Perennou C., Pritchard D. E., Rosenqvist A., Walpole M. (2015). State of the world’s wetlands and their services to people: A compilation of recent analyses.

[B6160901] Garvey James E., Stein Roy A., Thomas Heather M. (1994). Assessing how fish predation and interspecific prey competition influence a crayfish assemblage. Ecology.

[B6160028] Gaufin Arden R, Tarzwell Clarence M (1952). Aquatic invertebrates as indicators of stream pollution. Public Health Reports (1896-1970).

[B6159641] Gaufin Arden R, Tarzwell Clarence M (1956). Aquatic macro-invertebrate communities as indicators of organic pollution in Lytle Creek. Sewage and Industrial Wastes.

[B6160910] Gilinsky Ellen (1984). The role of fish predation and spatial heterogeneity in determining benthic community structure. Ecology.

[B6160792] Goss-Custard J. D. (1977). Optimal foraging and the size selection of worms by redshank, *Tringatotanus*, in the field. Animal Behaviour.

[B6161077] Guareschi S., Abellán P., Laini A., Green A. J., Sánchez-Zapata J. A., Velasco J., Millán A. (2015). ­­Cross-taxon congruence in wetlands: Assessing the value of waterbirds as surrogates of macroinvertebrate biodiversity in Mediterranean Ramsar sites. Ecological Indicators.

[B6161089] Halse S. A., Shiel R. J., Storey A. W., Edward D. H.D., Lansbury I., Cale D. J., Harvey M. S. (2000). Aquatic invertebrates and waterbirds of wetlands and rivers of the southern Carnarvon Basin, Western Australia. Records of the Western Australian Museum, Supplement.

[B6161146] Hellawell J. M. (1986). Biological indicators of freshwater pollution and environmental management.

[B6159555] Hodkinson Ian D., Jackson John K. (2005). Terrestrial and aquatic invertebrates as bioindicators for environmental monitoring, with particular reference to mountain ecosystems. Environmental Management.

[B6161101] Hornung Jonathan P., Foote A. Lee (2006). Aquatic invertebrate responses to fish presence and vegetation complexity in Western Boreal wetlands, with implications for Waterbird productivity. Wetlands.

[B6160769] James Daniel A., Csargo Isak J., Von Eschen Aaron, Thul Megan D., Baker James M., Hayer Cari-Ann, Howell Jessica, Krause Jacob, Letvin Alex, Chipps Steven R. (2012). A generalized model for estimating the energy density of invertebrates. Freshwater Science.

[B7434542] Johnston Thomas A., Cunjak Richard A. (1999). Dry mass-length relationships for benthic insects: a review with new data from Catamaran Brook, New Brunswick, Canada. Freshwater Biology.

[B6160946] Khamis K., Hannah D. M., Brown L. E., Tiberti R., Milner A. M. (2014). The use of invertebrates as indicators of environmental change in alpine rivers and lakes. Science of the Total Environment.

[B7434551] Krapu G. L., Reinecke K. J., Krapu G. L., Reinecke K. J. (1994). Ecology and Management of Breeding Waterfowl.

[B6160019] Krieger Kenneth A. (1992). The ecology of invertebrates in great lakes coastal wetlands: Current knowledge and research needs. Journal of Great Lakes Research.

[B6160991] Lavelle P., Decaëns T., Aubert M., Barot S., Blouin M., Bureau F., Margerie P., Mora P., Rossi J. -P. (2006). Soil invertebrates and ecosystem services. European Journal of Soil Biology.

[B6161005] Lawrence Andrew J., Soame Jane M. (2004). The effects of climate change on the reproduction of coastal invertebrates: Climate change and invertebrate reproduction. Ibis.

[B6160738] Lillie R. A., Evrard J. O. (1994). Influence of macroinvertebrates and macrophytes on waterfowl utilization of wetlands in the Prairie Pothole Region of northwestern Wisconsin. Hydrobiologia.

[B6159668] May R. M. (1990). How many species?. Philosophical Transactions of the Royal Society of London. Series B: Biological Sciences.

[B6160002] May R. M. (2010). Tropical arthropod species, more or less?. Science.

[B6159632] Ma Z., Cai Y., Li B., Chen J. (2010). Managing wetland habitats for waterbirds: an international perspective. Wetlands.

[B6160728] McCarthy Sarah G., Duda Jeffrey J., Emlen John M., Hodgson Garth R., Beauchamp David A. (2009). Linking habitat quality with trophic performance of steelhead along forest gradients in the South Fork Trinity River Watershed, California. Transactions of the American Fisheries Society.

[B6160055] Mora Camilo, Tittensor Derek P., Adl Sina, Simpson Alastair G. B., Worm Boris (2011). How many species are there on earth and in the ocean?. PLOS Biology.

[B7434594] Newton Ian (1998). Population limitation in birds.

[B6160719] Nudds T. D., Bowlby J. N. (1984). Predator–prey size relationships in North American dabbling ducks. Canadian Journal of Zoology.

[B7434577] Pöysä H., Elmberg J., Sjöberg K., Nummi P. (2000). Nesting mallards (Anas platyrhynchos) forecast brood-stage food limitation when selecting habitat: experimental evidence. Oecologia.

[B6160956] Prather Chelse M., Pelini Shannon L., Laws Angela, Rivest Emily, Woltz Megan, Bloch Christopher P., Del Toro Israel, Ho Chuan-Kai, Kominoski John, Newbold T. A. Scott, Parsons Sheena, Joern A. (2013). Invertebrates, ecosystem services and climate change: Invertebrates, ecosystems and climate change. Biological Reviews.

[B6161128] Sanders Mark D (2000). Enhancing food supplies for waders: inconsistent effects of substratum manipulations on aquatic invertebrate biomass. Journal of Applied Ecology.

[B7434507] Smock Leonard A. (1980). Relationships between body size and biomass of aquatic insects. Freshwater Biology.

[B6159546] Stork Nigel E., Eggleton Paul (1992). Invertebrates as determinants and indicators of soil quality. American Journal of Alternative Agriculture.

[B6161022] Strayer David L. (2006). Challenges for freshwater invertebrate conservation. Journal of the North American Benthological Society.

[B6160982] Strayer David L. (2010). Alien species in fresh waters: ecological effects, interactions with other stressors, and prospects for the future. Freshwater Biology.

[B6160679] Strayer David L., Dudgeon David (2010). Freshwater biodiversity conservation: recent progress and future challenges. Journal of the North American Benthological Society.

[B6160919] Taft Oriane W., Haig Susan M. (2005). The value of agricultural wetlands as invertebrate resources for wintering shorebirds. Agriculture, Ecosystems & Environment.

[B6903253] Thorp J. H., Covich A. P. (2009). Ecology and classification of North American freshwater invertebrates.

[B6903261] Thorp J. H., Rogers D. C. (2011). Field guide to freshwater invertebrates of North America.

[B7434516] Towers D. J., Henderson I. M., Veltman C. J. (1994). Predicting dry weight of New Zealand aquatic macroinvertebrates from linear dimensions. New Zealand Journal of Marine and Freshwater Research.

[B6159564] Vanni Michael J. (2002). Nutrient cycling by animals in freshwater ecosystems. Annual Review of Ecology and Systematics.

[B6160973] Vicente Filipe (2010). Micro-invertebrates conservation: forgotten biodiversity. Biodiversity and Conservation.

[B6160928] Weber L. M., Haig S. M. (1997). Shorebird–prey interactions in South Carolina coastal soft sediments. Canadian Journal of Zoology.

[B6160046] Zwarts L, Blomert A-M (1992). Why knot *Calidriscanutus* take medium-sized *Macomabalthica* when six prey species are available. Marine Ecology Progress Series.

